# Identify a Blood-Brain Barrier Penetrating Drug-TNB using Zebrafish Orthotopic Glioblastoma Xenograft Model

**DOI:** 10.1038/s41598-017-14766-2

**Published:** 2017-10-30

**Authors:** Anqi Zeng, Tinghong Ye, Dan Cao, Xi Huang, Yu Yang, Xiuli Chen, Yongmei Xie, Shaohua Yao, Chengjian Zhao

**Affiliations:** State Key Laboratory of Biotherapy and Cancer Center, West China Hospital, Sichuan University, and Collaborative Innovation Center for Biotherapy, Sichuan, P.R. China

## Abstract

The blood–brain barrier (BBB) is necessary for maintaining brain homeostasis, but it also represents a major challenge for drug delivery to the brain tumors. A suitable *in vivo* Glioblastoma Multiforme (GBM) model is needed for efficient testing of BBB crossable pharmaceuticals. In this study, we firstly confirmed the BBB functionality in 3dpf zebrafish embryos by Lucifer Yellow, Evans Blue and DAPI microinjection. We then transplanted human GBM tumor cells into the zebrafish brain, in which implanted GBM cells (U87 and U251) were highly mitotic and invasive, mimicking their malignancy features in rodents’ brain. Interestingly, we found that, although extensive endothelial proliferation and vessel dilation were observed in GBM xenografts, the BBB was still not disturbed. Next, using the zebrafish orthotopic GBM xenograft model as an *in vivo* visual readout, we successfully identified a promising small compound named TNB, which could efficiently cross the zebrafish BBB and inhibit the progression of orthotopic GBM xenografts. These results indicate that TNB is a promising BBB crossable GBM drug worth to be further characterized in human BBB setting, also suggest the zebrafish orthotopic GBM model as an efficient visual readout for the BBB penetrating anti-GBM drugs.

## Introduction

GBM (glioblastoma) is the most lethal intracranial brain tumor in adults and in recent years there is an significant ubiquitous upward trend in the incidence of GBM^1^. Despite surgery combined with chemotherapy and/or radiotherapy, the GBM patient’s prognosis is still not ideal. A number of innovative new treatments have also been recently evaluated in clinical trials, which include gene therapy, highly focused radiation therapy, immunotherapy and chemotherapies utilized in conjunction with vaccines^2–5^. However, we have to note that while some of these investigational treatments show promise, the most effective therapies introduced over the past three decades have only improved the median survival of GBM patients by an average of three months^2–5^.

Previous research showed that the degree of resection of the GBM tumor significantly affects the survival of patients^6^. However, GBM is characteristic of extremely aggressive growth and surgery is difficult to achieve complete resection of tumor tissue^2^. Thus, after surgical resection, further treatments with chemotherapy and radiotherapy are generally needed to kill the clinically undetectable GBM tumor cells. Unfortunately, although numerous experimental anti-GBM drugs have been tested in clinical trials, few showed satisfactory results^7^. This grim prognosis for GBM is at least partly due to the lack of successful drug delivery across the BBB. Advancement of knowledge in the molecular pathology underlying malignant transformation of brain tumor is rapidly expanding and is, to some extent, already assisting researchers in the design of tumor-tailored drugs. However, for designing of GBM drugs, we are still struggling to develop modalities to penetrate BBB and expose the entire tumor to such therapeutics at pharmacologically meaningful concentration.

BBB is essential for maintaining brain homeostasis and protecting the brain from toxic substances^8,9^. It is regulated by the proximity of a unique basement membrane and a tightly controlled molecular interaction between specialized subsets of cells including pericytes, astrocytes, microglia and neurons^10^. The major components of BBB include tetraspaning transmembrane proteins such as Claudins and the Occludin, as well as cytoplasmic anchoring proteins such as ZO-1^11^. The BBB serves roles in not only stopping circulating substances from entering the CNS (Central nervous system) but also facilitating and regulating the entry of many substances that are critical to CNS function. Given the functional and structural complexity of the BBB, efficient and accurate BBB crossable drug screening is necessary to be carried out in *in vivo* systems. Previous studies of the BBB development and maturation in zebrafish have shown that the zebrafish indeed gradually develop a sophisticated BBB with tight-junction proteins and active transport systems, which is functionally and structurally similar to that of higher vertebrates. These studies suggest that zebrafish could be applied as an experimental model organism for BBB-penetrating drug screening^11–13^.

On the other hand, as embryo–larval zebrafish lack a fully functional adaptive immune system until 28 days after fertilization (dpf), which makes it possible to implant human tumor cells without rejection^14–16^. Recently, researchers have proved that the human glioma cells can robustly grow, migrate in the brain of embryo-larval zebrafish. Interaction of implanted glioma cells with the microglia cells was also determined^17^. Furthermore, using this zebrafish tumor model, Wehmas *et al*. observed significant tumor inhibition effect with exogenous compound (a selective PI3K inhibitor-LY294002) treatment, which was similar to that observed in organotypic mouse brain tissues^18,19^. As a model species, zebrafish has numerous strengths, including offspring number, *in vitro* fertilization and embryo transparency^20^. Here, a particular advantage of the zebrafish xenograft model is that only a small number of cancer cells (50–500 cells per fish) are required for xenotransplantation. All these results strongly indicate the embryo-larval zebrafish can be applied as an experimental tumor xenografted model for efficient BBB-penetrating drug identification^11^.

In the present study, we successfully established human GBM orthotopic xenograft model in 3dpf flk:eGFP transgenic zebrafish using U87 and U251 human glioblastoma cells. Interestingly, with GBM micro-xenografts growing in the zebrafish brain, we found that the cerebral capillaries dilation and brain microvascular endothelial cells (BMECs) proliferation were intensive, however, the functionality of BBB in the GBM xenografts were generally not disturbed. At last, using this zebrafish orthotopic GBM model, we discovered one of our previously synthesized compounds—TNB, which could readily cross the zebrafish BBB and inhibit the growth of GBM xenografts in the brain. Thus, our data indicates that zebrafish orthotopic GBM model is an effective tool for testing BBB penetrating drugs and TNB might present as a promising BBB crossing drugs for GBM.

## Materials and Methods

### Zebrafish strains

Lines used in this study included flk: eGFP (zfin: s843Tg), flk: mCherry (zfin: zf527Tg), AB and Casper mutant (originally developed in Zon laboratory at Harvard Medical School). All fish were bred and maintained normally (temperature, 28°C; pH 7.2–7.4; 14 h on and 10 h off light cycle)^20^. Except Casper mutant line, all the zebrafish embryos that developed beyond 24 hours post-fertilization (hpf) were treated with phenylthiourea (PTU; 0.003%, w/v; Sigma, USA). All animal experiments were performed according to the guidelines of the Animal Care and Use Committee of Sichuan University (Chengdu, Sichuan, China) and approved by the institutional review board of the Medical Faculty at the West China Hospital, Sichuan University.

### Cell culture and establishment of a stable red fluorescent protein and green fluorescent protein expressing GBM cell lines

U87, U251 and 293 T cells were obtained from the American Type Culture Collection (ATCC) and maintained in Dulbecco’s modified Eagle’s medium (DMEM) (Gibico, USA), containing 10% fetal bovine serum (FBS) (Caoyuanlvye, China) and 1:100 Pen/Strep (Invitrogen, USA) at 37 °C with 5% CO_2_. To establish stable RFP-expressing or GFP-expressing U87, U251 and 293 T cell lines, wild-type cells were infected with lentivirus containing a CMV driving GFP or RFP gene. FACS (Aria, BD) was applied to sort the pure fluorescent protein expressing cells 48 hours post infection. The stable RFP and GFP expressing cells were then cultured in normal conditions.

### Microinjection of GBM cells

The RFP and GFP-expressing U87, U251 and 293 T cells were washed, re-suspended in basic DMEM. For microinjection of tumor cells into the embryos, the concentration of cell is 1 × 10^7^ cells/ml and this mixture was loaded into a borosilicate glass needle pulled by a Flaming/Brown micropipette puller (Narishige, Japan, PN-30). By using an electronically regulated air-pressure microinjector (Harvard Apparatus, NY, PL1–90)^21^, 5~10 nanoliters suspension containing about 200–500 cells were injected into the brain of Tg(flk:eGFP) or Tg(flk:mCherry) zebrafish embryos (3dpf), which were anesthetized with 0.04 mg/ml tricaine (MS-222; Sigma, USA). After injection, zebrafish were washed twice with fresh fish water and examined for the presence of fluorescent cells. After each implantation, about 30 fish were selected and cultivated in 6-well plate containing 2 ml of fish water containing with Pen/Strep (1:100) at 33 °C and subsequently documented photographically. Fish water was changed daily, and larva that more than 7 days old were fed twice a day with grinded brine shrimp.

### Caudal vein microinjection of dextran, DAPI, doxorubicin and TN-B

The dextran (MW: 70000; Sigma, USA), DAPI (MW: 350; Sigma, USA), Lucifer Yellow (MW: 457; Sigma, USA), Evans Blue Dye (MW:960; Sigma, USA) doxorubicin HCl (ADR) (Bedford, OH, USA) and TNB were loaded into a borosilicate glass needle pulled by a Flaming/Brown micropipette puller (Narishige, Japan, PN-30). After anesthetized with 0.04 mg/ml tricaine (MS-222; Sigma, USA), 1–3 nanoliters suspension was injected into a zebrafish embryo from caudal vein using an electronically regulated air-pressure microinjector (Harvard Apparatus, NY, PL1-90)^21^. Injected zebrafish were washed twice with fresh fish water and imaged ~30 min later.

### Imaging

For live imaging of zebrafish, selected embryos were mounted in low melting point (LMP) agarose (1%, wt/vol) at the bottom of a 29 mm glass bottom dish and covered with fish water at 33 °C. Digital micrographs or real-time tracking images were taken either with a Zeiss Imager.Z1 fluorescence microscope (Carl Zeiss, Germany) or a Leica SP5 resonant scanning confocal (Leica, Germany).

### Statistical Analysis

Data was assayed by unpaired student’s t test using SPSS18.0 statistical analysis software. A level of *P < 0.05 was regarded as statistically significant.

## Results

### Verification of functional BBB in 3dpf zebrafish embryo

**Figure 1:**
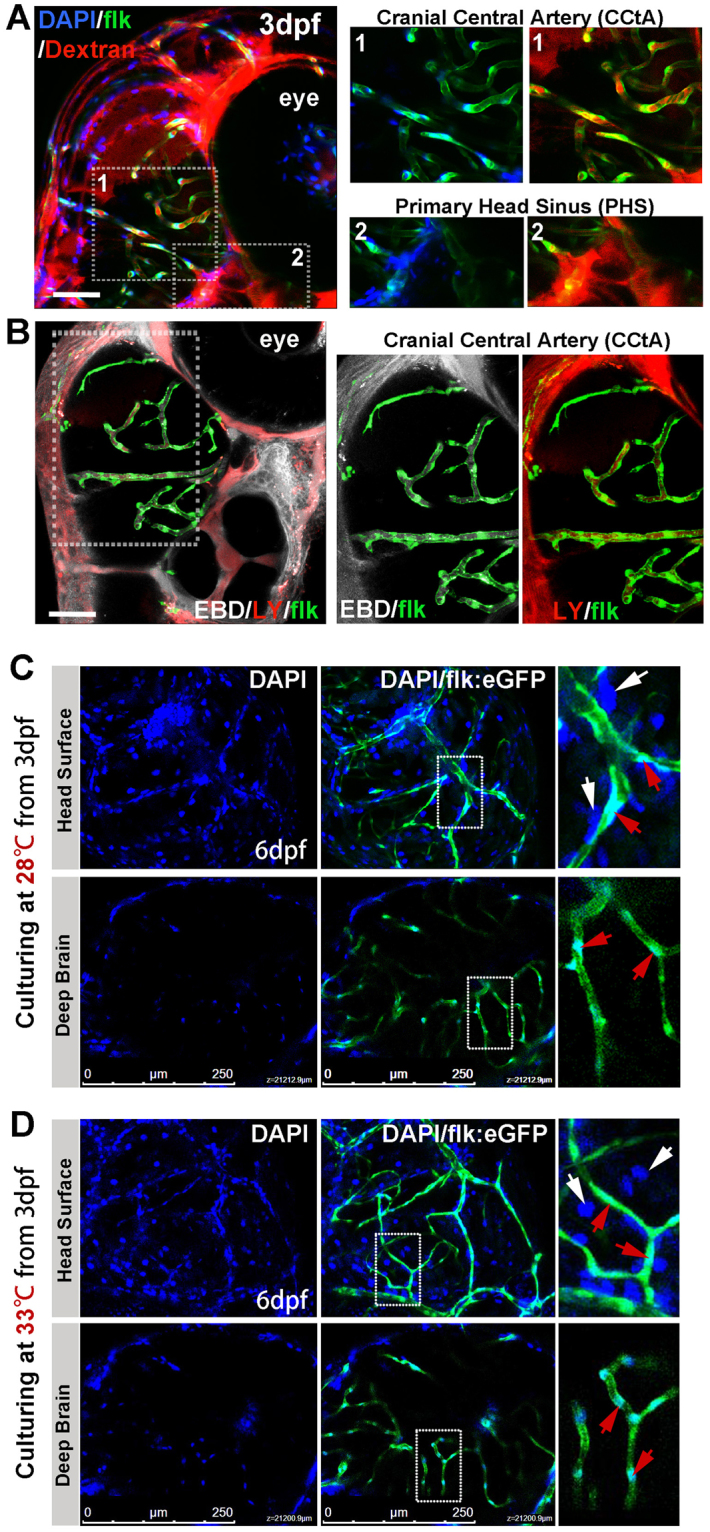
Imaging of functional BBB in 3dpf and 6dpf zebrafish. (**A**,**B**) The Dextran Texas Red (70,000 MW, 1 mg/ml), Evans Blue Dye (EBD, 961 MW, 5 mg/ml), Lucifer Yellow (LY, 457 MW, 4 mg/ml) and DAPI (350 MW, 1.5 mg/ml) were mixed and injected into the circulation of 3dpf flk:eGFP or flk:mCherry embryos (5 nl/fish, green indicating endothelial cells) from caudal vein (CV). Z-stack confocal living images of the cerebral capillaries and segmental capillaries (Fig. S2A) were obtained at 30–60 min after injection. (**A**, area1) Dextran and DAPI were restrained in cerebral capillaries (CCTA), but were leaked out from primary head Sinus (**A**, area2) and segmental capillaries (Fig. S2A). (**B**) CBD (white) and LY (red, pseudocolor) were restrained in CCTA (mCherry was pseudocolored as green). Areas in dotted boxes were magnified right. (**C**) DAPI was injected into the circulation of 6dpf embryos (maintained at 28 °C) from CV. Z-stack confocal living images were taken at 1 h post injection. White arrows indicating nucleus of host cells neighboring to vessels; Red arrows indicating endothelial nucleus. (**D**)DAPI was injected into the circulation of 6dpf embryos (normally maintained 3dpf zebrafish were shifted to 33 °C for 3days) from CV. Z-stack confocal living images were taken at 1 h post injection. White arrows indicating nucleus of host cells neighboring to vessels; Red arrows indicating endothelial nucleus.

It was reported that the endothelial tight junction-based BBB of zebrafish is functionally and structurally similar to that of higher vertebrates^10,22,23^. To study the interaction between the glioblastoma and local BBB in zebrafish brain, we first verified the existence of functional BBB in zebrafish embryos. As the BBB function is related to its permeability to compounds of different molecular weight^24–26^. DAPI (blue fluorescent, MW = 350)^13,27^, Evans Blue Dye (EBD, 961 MW), Lucifer Yellow (LY, 457 MW) and Dextran Texas Red (red fluorescent, MW = 70,000) was microinjected into the circulation of zebrafish from caudal vein to intuitionally prove the existence of functional BBB in 3dpf (days post fertilization) flk:eGFP/mCherry zebrafish embryos (eGFP or mCherry marks all endothelial cells in blood vessels, and endothelial precursors cells in the blood circulation after 3dpf). As expected, about 1hr post the microinjection, most of the cells in the zebrafish segments, including endothelial cells, muscle cells and cells in spinal cord were stained blue in the nucleus, and with some red dextran leaked into the tissue surrounding the blood vessels (dorsal aorta, segmental capillaries) (Fig. S2). However, at the same time in the zebrafish brain, all four fluorescent tracers (DAPI, Dextran, LY and EBD) were strictly restrained in the cerebral capillaries, only the nucleus of BMECs were stained blue (Fig. 1A, area1), suggesting the existence of functional BBB in 3dpf zebrafish brain.

Zebrafish are usually maintained at 28 °C, but it has been reported that the development of zebrafish were normal even the temperature was up to 34 °C^28^. Using zebrafish as tumor xenograft model, to compromise between the optimal temperature requirements for fish and mammalian cells (37 °C), in this study, zebrafish embryos were generally maintained at 33–34 °C^29,30^. To test the possible alteration of BBB function in the zebrafish embryos culturing at 33 °C, zebrafish embryos were transferred to 33 °C incubator and cultured for 3days, then DAPI was injected into the zebrafish (33 °C, 3dpf) circulation from caudal vein. As expected, although the embryos at 33 °C developed a little faster than those maintained at 28 °C, confocal Z-stack images showed that DAPI were still completely restrained in the cerebral capillaries (Fig. 1C,D), suggesting that the BBB function of zebrafish embryos was not affected when culturing at 33 °C.

### Establishment of orthotopic GBM xenograft model using flk:eGFP zebrafish embryos

Based on previous zebrafish tumor model^23,31,32^, we extended our study to establish an orthotopic xenograft zebrafish model with human malignant glioma cell lines, U87 and U251.

To take advantage of the transparency of Casper mutant zebrafish, by outcross and incross Casper mutant line with flk:eGFP zebrafish, we established the flk:eGFP Casper zebrafish, which are transparent even into adulthood and the vascular endothelial cells are GFP labeled.

**Figure 2:**
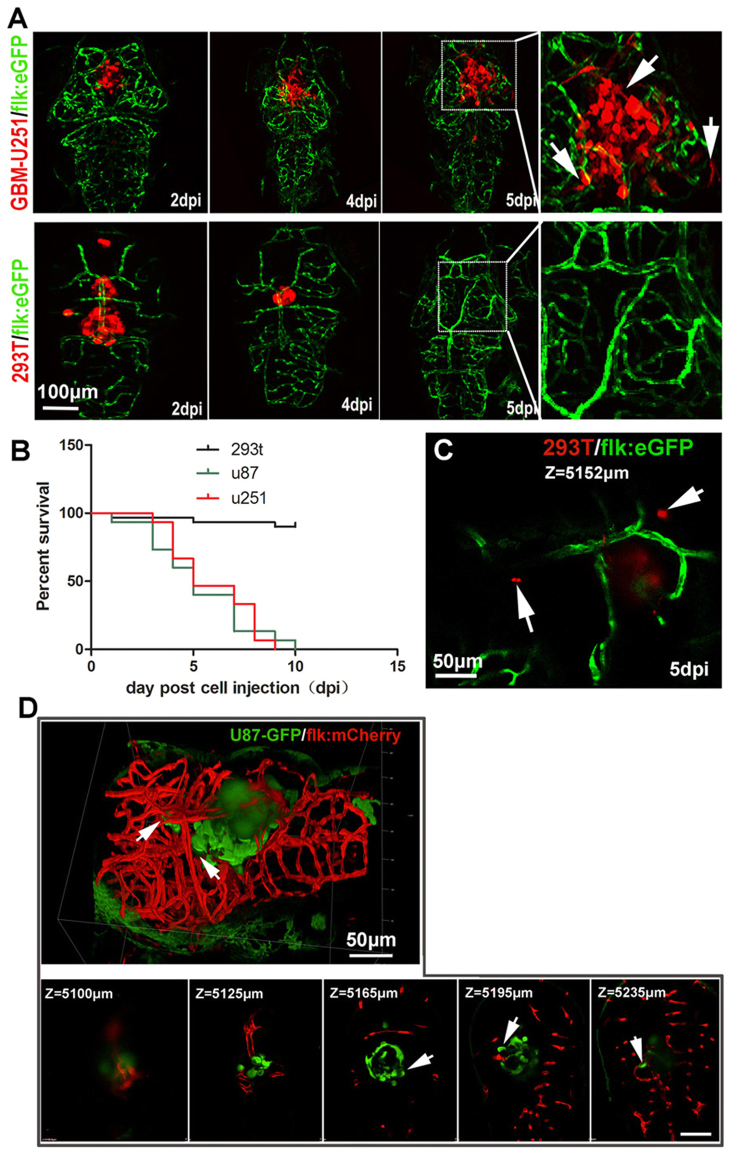
Establishment of GBM xenografts in flk:eGFP Casper zebrafish. (**A**,**B**) 200–500 GBM tumor cells (U87-RFP, U251-RFP) were intracranial implanted into 3dpf flk:eGFP Casper zebrafish and monitored by confocal microscope every 24 hours, 293T-RFP cells implantation was set as control. Areas in dotted boxes were magnified right, arrows indicating infiltrating tumor cells. (**B**) The survivorship curve of U87 and U251 tumor-bearing zebrafish and control 293 T injected zebrafish. (**C**) A high-resolution image of intracranially implanted 293t-RFP cells at 6dpf, arrows indicating the cell debris. (**D**) A reconstructed 3D image of a U87-GFP xenograft in the deep brain of flk:mCherry zebrafish at 6dpf, arrows indicating infiltrating tumor cells. Z-stack serial images show the depth of the GBM xenograft in zebrafish brain, arrows indicating infiltrating tumor cells.

After injection of 200–500 U87-RFP or U251-RFP cells into the 3dpf Casper flk:eGFP zebrafish brain, the growth of the xenografts in zebrafish recipients were tracked using a confocal microscope (Leica SP5). For the U87 intracranial xenografts, some of the injected tumor cells died within the first 2 days, which resulted totally 20–35% tumor volume reduction (Fig. S1). However, the survived U87 tumor cells generally regrown orthotopic from day3 until the death of recipients, showing little infiltrative growth pattern (Fig. S1). For the U251 intracranial xenografts, initial cell loss was not detected and implanted tumor cells kept growing in the fish brain from the first day after implantation. Generally, the U251 cells were showing an extensive infiltrative growth pattern into the deep brain parenchyma (Fig. 2A), similar to that observed in rodent xenograft model and the clinical features of human GBM. As a control, 293T-RFP cells (200–500cells/fish) were also injected into the zebrafish brain, different to that observed in GBM tumor cells transplantation, injected 293 T cells died away gradually from the cerebrum within 2 days (Fig. 2C). The survival analysis results show that most of the zebrafish recipients with tumor xenografts (U87 or U251, n = 45 for each group) died within 10 days, while those transplanted with 293 T cells survived until the termination of our experiments (Fig. 2B). To prove that the injected tumor cells were actually infiltrated into the deep brain, we took a 3D Z-stack image of the whole brain of zebrafish with U87-GFP xenograft (Fig. 2D). The Z-stack serial images indicated that the U87-GFP tumor cells were infiltrated deeply in the zebrafish brain (depth is around 180 μm for a xenograft in the brain, the diameter of a 6 dpf zebrafish brain is around 300 μm) (Fig. 2D, Z-stack serial images).

### Implanted human GBM cells were active in the zebrafish brain

**Figure 3:**
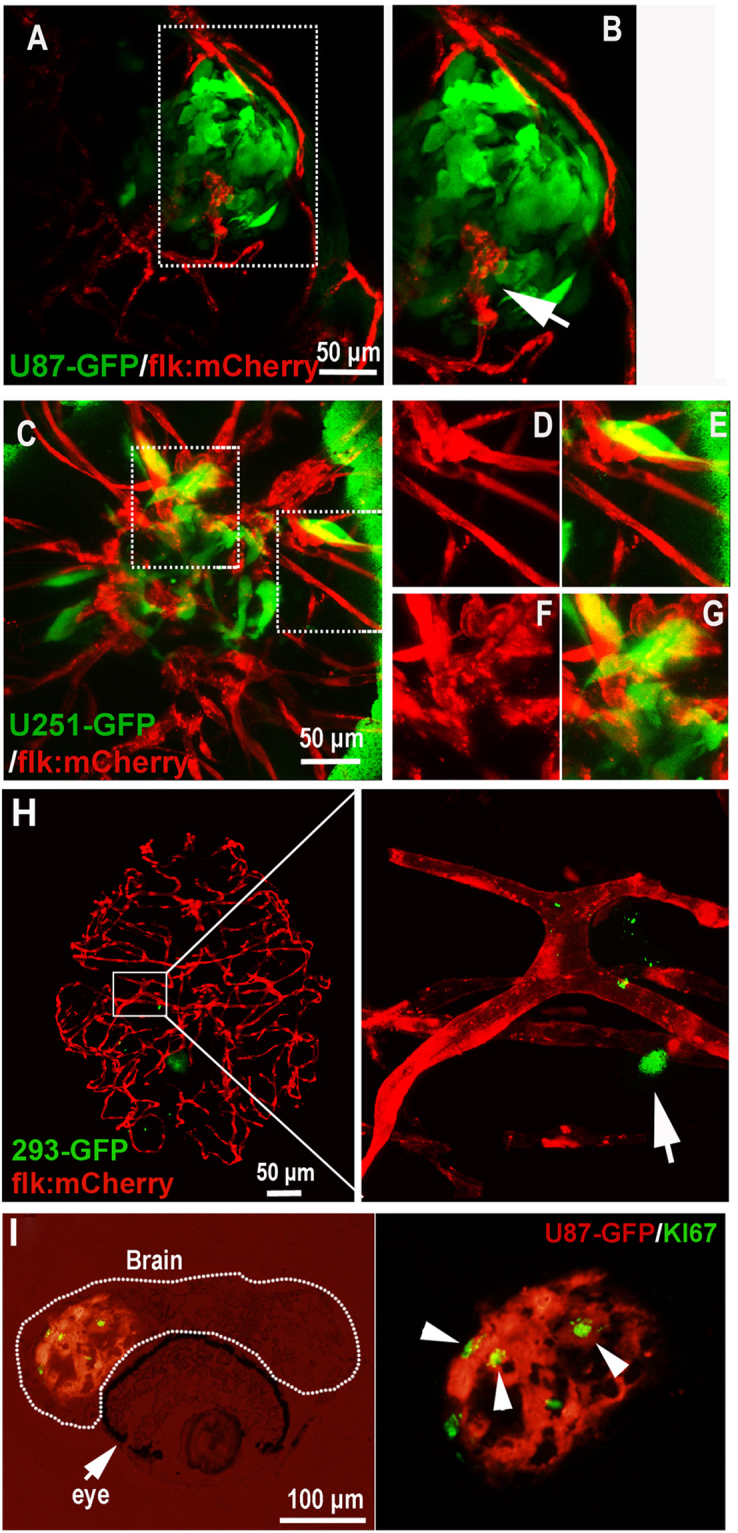
Active interaction between GBM xenografts and cerebral capillaries. (**A**,**B**) U87-GFP xenografts induced angiogenesis from neighboring host vessel within the tumor xenograft at 5dpi, area in dotted box was magnified in B, and arrow indicating the endothelial sprouts. (**C**–**G**) Tumor induced angiogenesis (**F**,**G**) and infiltrative tumor growth (**D**,**E**) of U251-GFP xenografts at 5dpi were detected in the zebrafish brain. (**H**) Representative image showing the 5dpi zebrafish brain with 293-GFP cells (arrow)(200–500 293-GFP cells were implanted into the brain of 3dpf zebrafish); implanted 293-GFP cells failed to survive in the brain parenchyma and cerebral capillaries were not affected by 293-GFP cells. (**I**) Immunostaining of U87 xenograft in zebrafish brain indicating the implanted tumor cells were proliferating (ki67+), arrowheads indicating the GFP+/ki67+ tumor cells in the xenograft in zebrafish.

Due to the robust induction of tumor angiogenesis by U87 cells, U87 cell line receives significant attention in GBM study, especially in assessing tumor vasculature formation and anti-vasculature therapies^8,33^. Similarly to the observation in rodent xenograft model, U87 xenografts in zebrafish could induce angiogenesis efficiently. Using the high-resolution confocal microscope, we found the neovessels in the GBM tumor mass were tortuous and not as smooth as the surrounding host vessels (Fig. 3A,B). U251 GBM cell line is well known to recapitulate the infiltrative growth features of human GBM and has been widely used in xenogeneic mouse models. Here, in our zebrafish U251 xenograft model, implanted U251 cells showed an infiltrative pattern of growth into normal brain parenchyma (Fig. 3C–G), similar to that observed in rodent and human. With the help of confocal microscope, we could even evaluate the infiltrative growth and tortuous microvasculature of GBM xenograft in zebrafish at single-cell level. At last, to further support our observation, we evaluated the health of the injected tumor cells. Dual-color immunostaining for the GFP (U87-GFP xenograft) and ki67 was applied and ki67+/GFP+ tumor cells were constantly detected within the orthotopic xenograft (Fig. 3H), indicating a proliferating status of the implanted tumor cells.

### Interaction of GBM tumor cells with the BBB in zebrafish brain embryo

**Figure 4:**
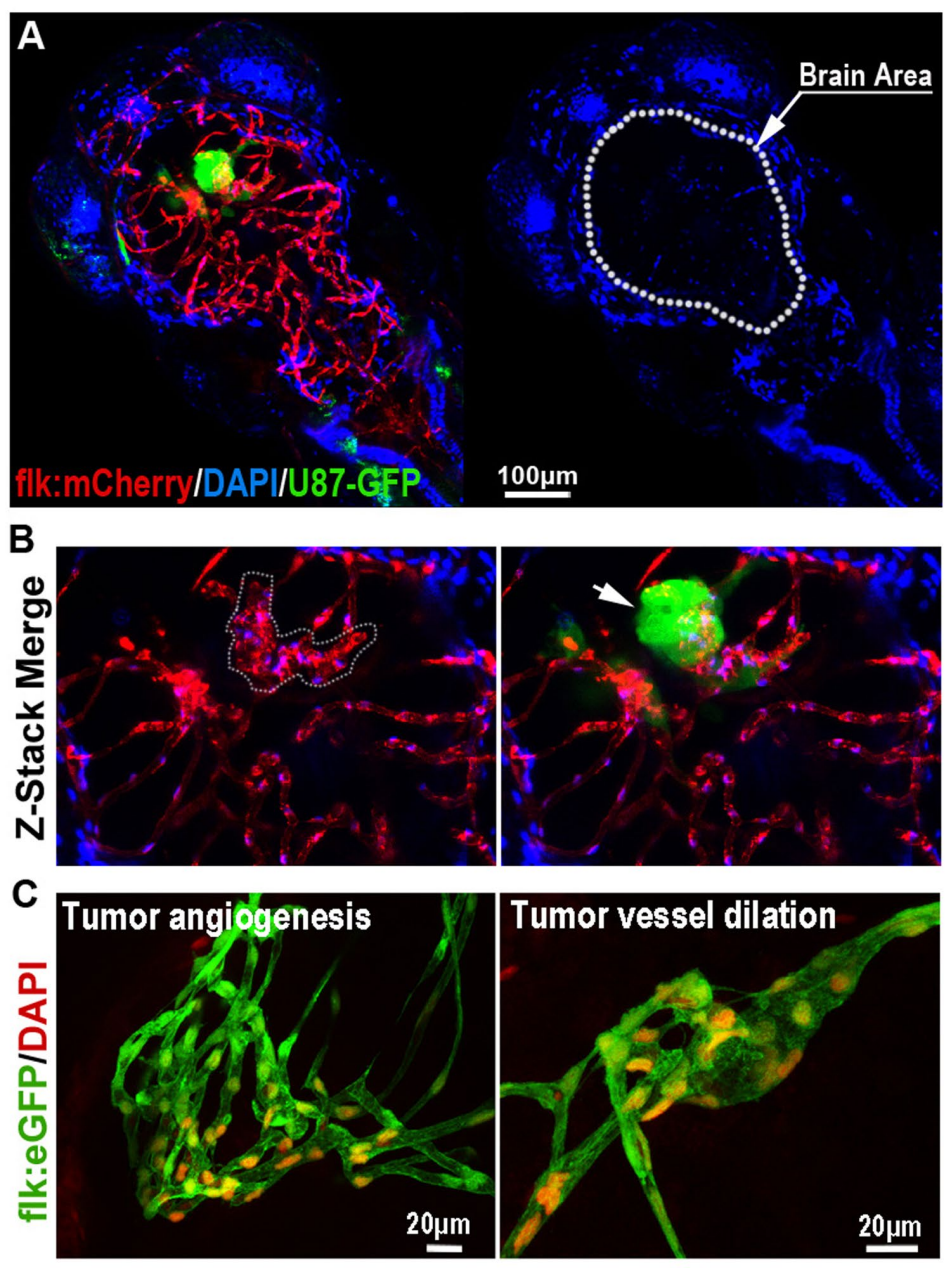
The Blood-brain Barrier is general not disturbed by GBM xenografts. (**A**) Z-stack confocal images showing the location of GFP-labeled GBM xenograft in a 6dpf zebrafish, and the dilation of tumor-related red cerebral capillaries. DAPI was injected into the zebrafish circulation 1 hour before taking the living image. Dotted line indicates the brain area. (**B**) Higher resolution image of the GBM xenograft showing the obvious vessel dilation and related endothelial proliferation (indicated by nucleus number) within the tumor (arrow). (**C**) DAPI was strictly restrained in the tumor vessels of GBM xenografts in 7dpf zebrafish brain. Endothelial nucleus of the GBM cerebral vessels were stained by DAPI (red, pseudo-color), no cells that outside of the vessels were stained, indicating the incapability of DAPI crossing the GBM vessels.

As intensive interaction between the GBM xenograft and cerebral capillaries was observed, we next tried to characterize the possible BBB alteration in the GBM-implanted zebrafish brain. To study the influence of growing GBM microtumor on the cerebral capillaries in zebrafish brain, we injected U87-GFP tumor cells into the flk:mCherry zebrafish brain (3dpf) as previously described. 3 days later, DAPI was injected into the GBM-implanted zebrafish circulation to test the function of BBB. Interestingly, with the co-option of GBM tumor cells, hyper proliferation of BMECs in the cerebral capillaries were generally detected, which resulted in the tortuosity and dilation of these capillaries (Fig. 4A,B). Surprisingly, DAPI tracer injection showed that the tortuous cerebral capillaries were still able to hold the DAPI, indicating the intact of BBB (Fig. 4C). However, occasionally, we found just a few tumor cells (even one tumor cell) were sufficient to cause DAPI leakage (Fig. S3). These results suggest that, similar to that in mammals^34^, invading GBM tumor cells in the zebrafish brain tend to actively interact with the brain capillaries, causing a complex and unstable status of GBM related BBB.

### Identify BBB penetration pharmaceuticals in living zebrafish using confocal

**Figure 5:**
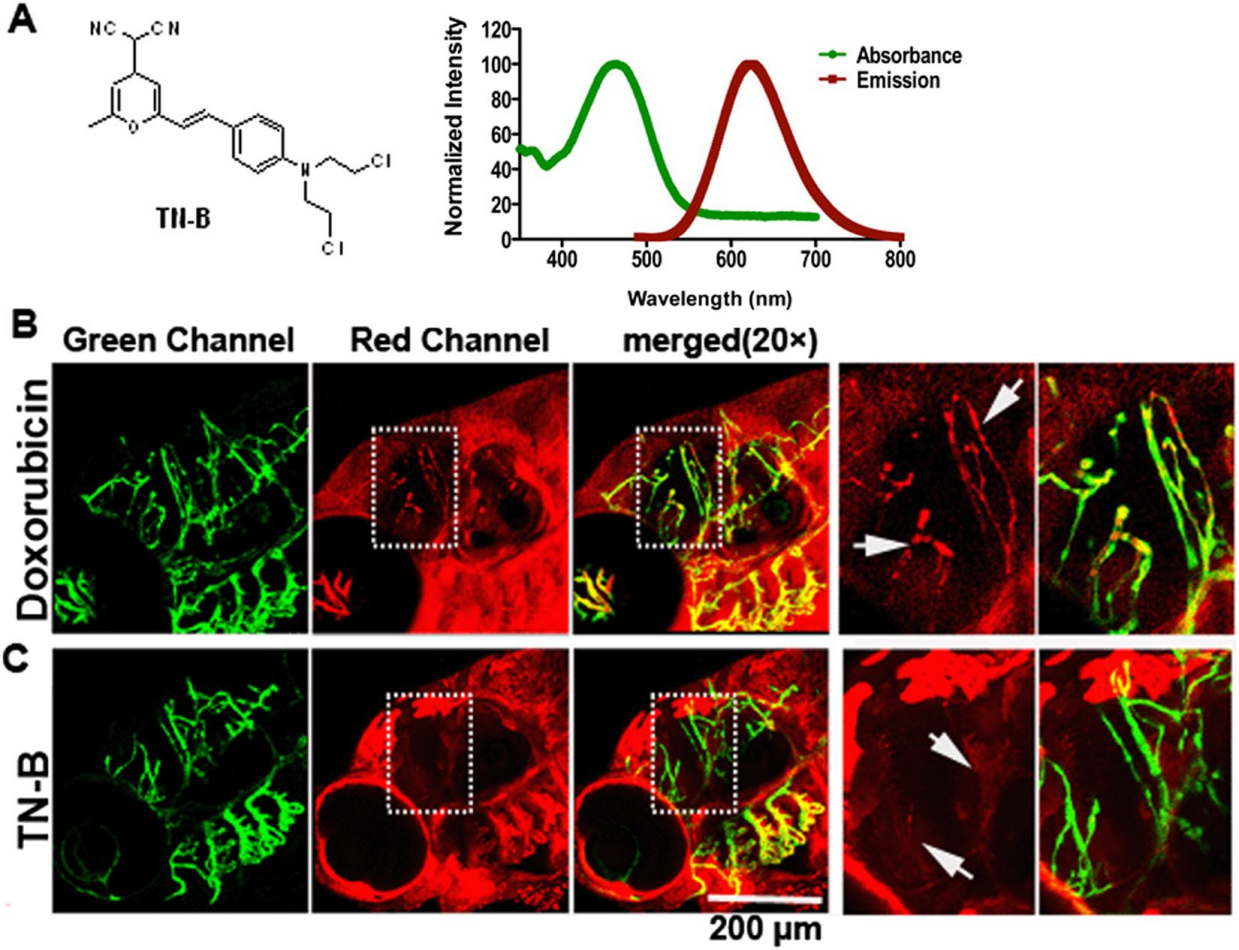
TN-B is capable of crossing the zebrafish BBB efficiently. Doxorubicin (Adriamycin®) and TN-B were injected into the circulation of 6dpf flk:eGFP zebrafish through caudal vein. Living images were obtained about 30 min after injection. (**A**) The structure of Nitrogen mustard based TN-B (left panel, A). The absorbance spectrum (green bold line) and emission spectrum (red bold line) of TN-B are shown (right panel, A). (**B**) Doxorubicin (Red Fluorescence) was completely restrained in cerebral capillaries, area in dotted box was magnified right, Doxorubicin was restrained in cerebral capillaries (arrows in **B**). (**C**) TN-B (showing with red fluorescence) readily penetrated the BBB into the brain parechyma, areas in dotted boxes were magnified right, TN-B crossing the cerebral capillaries into brain tissue (arrows in **C**).

These results raised the possibility that zebrafish may represent as an experimental model organism to identify pharmaceuticals that are able to penetrate the BBB and target GBM by direct live imaging. To test the hypothesis, following the principles of BBB free-diffusion chemicals and referring to the previous reported BBB penetrable drugs or probes^35^, we designed and synthesized a BBB penetrating compound library^36^. Using transparent zebrafish embryos and Z-Stack confocal system, we identified a nitrogen mustard-based DNA cross-linking small chemical (TNB) that could penetrate the BBB readily in the zebrafish brain (Fig. 5A). Doxorubicin (Adriamycin®), a traditional chemotherapy drug, which has long been proven incapable of crossing the patient’s and animal’s BBB was set as a negative control. Both Doxorubicin and TNB were injected into the zebrafish embryos blood circulation (~5 nanoliters, 1 mg/ml) through caudal vein. As expected, the red fluorescent Doxorubicin freely diffused out from the blood vessels in the head surface, while complete restrained in the cerebral vessels (CtA) (Fig. 5B). In contrast, for the TN-B, about 1 h post injection, the vascular margin of CtA was completely disappeared and the adjacent brain tissue was stained red, suggesting the readily crossing of TN-B through the zebrafish BBB (Fig. 5C).

### TNB could efficiently inhibit the progression of GBM tumors in the zebrafish brain

**Figure 6:**
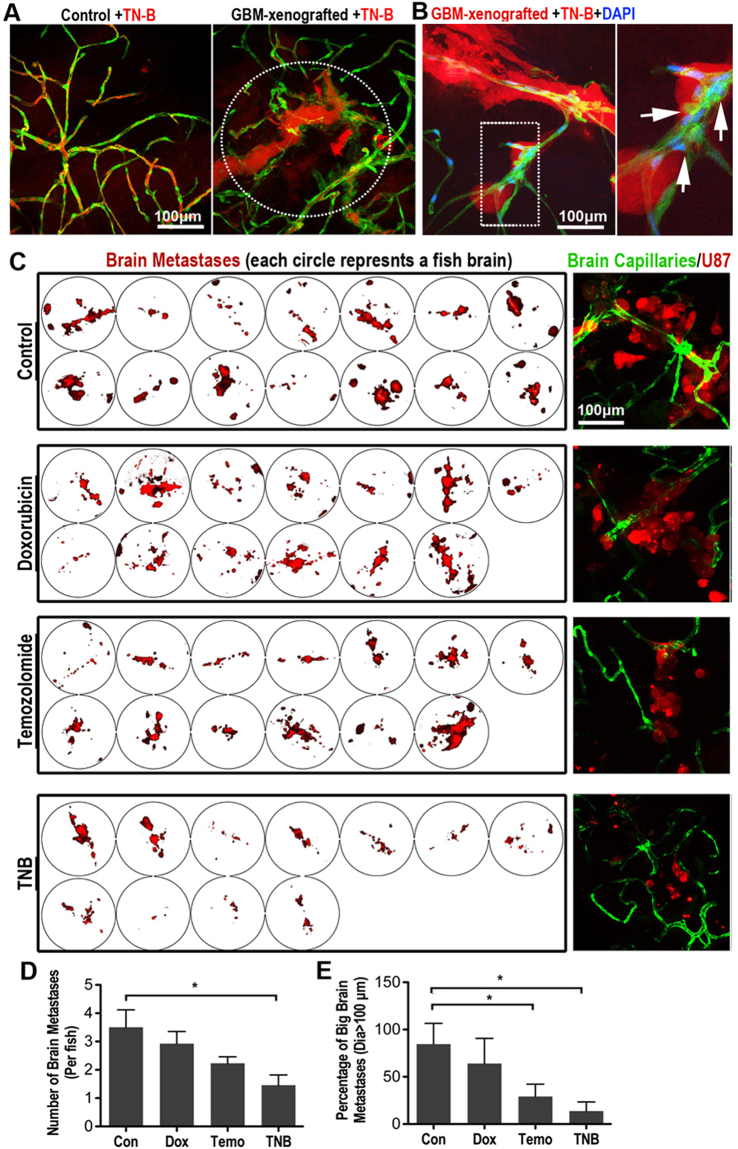
TN-B inhibits the invasion and growth of GBM xenografts in zebrafish brain. (**A**) TN-B was directly injected into the circulation of 5dpi zebrafish with or without GBM xenografts. Confocal images were taken 1 hour post tracer and drug injection. Dotted circle indicating the tumor area. (**B**) TN-B and DAPI was injected into the circulation of zebrafish (with 5dpi red GBM xenograft). Confocal images at 8 hours post injection showed the DAPI was still hold in cerebral capillaries (Arrows), indicating the BBB was not destroyed by the TN-B treatment. Area in dotted box was magnified right. (**C**) Drugs (Doxorubicin, Temozolomide, TN-B and control DMSO) were directly added into the culture medium of living zebrafish embryos (4dpf), which were implanted with GBM-U87 at 3dpf as previously described (n = 20 in each group). After 3days treatment, all the brains of zebrafish embryos were imaged with confocal microscope. Left panels show the intracranial GBM metastases (red) in each zebrafish brain. Right panels show representative images of the intracranial GBM cells (red) and cerebral capillaries (green) with high resolution. (**D**,**E**) Quantitative analysis of the number of whole GBM metastases or relative big GBM metastases in zebrafish brain with or without drug treatment at 7dpf, diagrams showing means and SEM.

Our experimental results indicate the nitrogen mustard-based DNA cross-linking small chemical TN-B as a potential small compound for the treatment of GBM. Firstly, to test the distribution of TNB in the zebrafish brain with GBM xenograft, we injected TN-B (~5 nanoliters, 1 mg/ml) directly into the zebrafish circulation through the caudal vein. Interestingly, confocal imaging showed that relatively more TN-B was pumped into the tumor xenograft area, possibly due to the dilation and higher permeability of the tumor-associated vessels (Fig. 6A). Furthermore, to investigate the possible direct toxic effect of TN-B on the BBB permeability, TNB was injected into the zebrafish (with 5dpi GBM xenograft) circulation together with DAPI and further cultured for 8 hours. Similar to our previous observation, at the fringe of the tumor mass, hyper proliferation of ECs was detected, however, the DAPI tracer was still strictly restrained in these cerebral capillaries, suggested that the functionality of BBB was not disturbed by TN-B treatment (Fig. 6A,B).

Then, to test the possible effect of TN-B on the GBM *in vivo*, zebrafish GBM-U87 xenograft model was applied. One day post tumor cell injection, zebrafish embryos with robust GBM xenografts were selected out and divided into 4 groups (n = 20 in each group). Doxorubicin (2 μM), Temozolomide (100 μM) and TN-B (10 μM) were directly added into the embryo culture medium, DMSO was set as control. Culture medium and drugs were changed every 24 hours for a consecutive 3-day treatment. Each zebrafish embryo brain was then imaged and measured using confocal. Confocal images and quantitative analysis results showed that both the number (Fig. 6C,D) and size (Fig. 6C,E) of brain tumors in the TN-B and Temozolomide treated zebrafish were dramatically reduced, comparing with the DMSO control and Doxorubicin group. These *in vivo* results indicated TNB as a potential drug for the GBM treatment. However, we notice that, although TN-B could efficiently inhibit the progression of GBM xenograft in the zebrafish brain, 3-day TNB treatment resulted in a little higher animal death rate comparing with that in other groups (TN-B 8/20; DMSO 4/20; Doxorubicin 7/20; Temozolomide 7/20), which suggested a possible higher system toxic of TN-B to the recipients. Altogether, these results indicate that zebrafish GBM xenograft model represents as a novel *in vivo* visual readout for BBB penetrating GBM drugs and TN-B might be a potential BBB penetrating anti-GBM drug.

## Discussion

GBM is the most common primary brain tumors, and the aggressive invasion by malignant glioma cells into surrounding normal brain tissues has been recognized as an important cause for relapse after surgical excision^37^. Despite the improvement in therapeutics in recent years, the median survival time for glioblastoma is still no more than 14 months after diagnosis^38^. The impermeability of the BBB is a major obstacle for drug delivery to the invading GBM tumor cells in the deep brain tissue. Designing an efficient and reliable animal model to screen the BBB penetrating drugs is urgent.

It is well known that BBB has its complexity. The major components of BBB not only include astrocytes and brain capillary endothelial cells but also many tetraspaning transmembrane proteins, and it has proven difficult to recapitulate the complexity *in vitro*. Thus, testing for BBB-modulating drugs has been a slow, hit-or-miss process that requires living animals. Here, we reported an efficient and reliable BBB-penetrating drug screening animal model, in which we could not only directly observe the penetrating process of testing drugs, but also track the response of GBM tumor cells outside of the brain capillaries.

Previous *in vitro* systems for studying the BBB have suffered from numerous shortcomings, including poor scalability and inadequate separation of blood and brain compartments. Our and others’ studies have proved that the BBB of zebrafish is functionally and molecularly similar to that of higher vertebrates^11,39–41^. Expression of claudin-5 and ZO-1 has been detected in cerebral microvessels of larval zebrafish from 3 dpf. Relative to the ABC transporter genes in zebrafish, such as P-gp (ABCB subfamily), BCRP (ABCG subfamily), MRPs (ABCC subfamily), previous studies have characterized the expression pattern and functions of these ABC transporters in zebrafish embryos, indicating efflux transporters in the BBB may function in zebrafish in a similar manner to those of higher vertebrates. While similarities exist between the zebrafish and mammalian BBB, the combination of zebrafish-based assays could generate useful information regarding the structure and permeability of BBB. ABC transporter genes are big family genes, and zebrafish don’t express all the paralogous genes of human’s. In addition, although peptide alignment analysis shows some of the zebrafish ABC transporter genes are similar to that of human’s, the functions of these genes in zebrafish still need to be further determined. We need to be very careful when evaluating the permeability data of drugs using zebrafish BBB model. Of course, such results need to be further determined in human BBB setting, yet they could be very helpful to the development of clinical drugs and imaging GBM-targeting agents in preclinical studies.

It has been reported that the invading GBM tumor cells tend to coopt with the brain capillaries. Furthermore, the interaction of tumor cells with the surrounding neuron, microglia and endothelium, together with the angiogenic secretion property of the malignant cells, make a complex and unstable status of GBM related BBB^34^. For our zebrafish xenograft model, only a small number of GBM tumor cells (200–500 cells per fish) are injected into the recipient’s brain, which better simulates earlier stages of GBM progression. However, similar to that observed in previous mouse model, implanted tumor cells are able to induce tumor angiogenesis and generate new vessels in the central glioma xenografts, which are believed to lack tight-junction and BBB function^42^. For the invasion of these implanted tumor cells, they generally migrate along the cerebral capillaries, similar to that observed in rodent and clinical patients. Interestingly, with the co-option of GBM tumor cells, hyper proliferation of BMECs in the cerebral capillaries were generally detected, which resulted in tortuosity and dilation of these capillaries. Surprisingly, DAPI tracer injection showed that the tortuous cerebral capillaries were still able to hold the DAPI, indicating the intact of BBB. However, occasionally, we found just a few tumor cells (even one tumor cell) were sufficient to cause DAPI leakage. These results suggest that, as previous studies have demonstrated, invading GBM tumor cells are able to breach the BBB. However, the BBB of most tumor cells co-opted cerebral capillaries are still intact, although they are hyper proliferated and tortuous. We believe that there must be an unidentified repair mechanism of maintaining the function of BBB in the glioma tumor invading context.

A major concern for the zebrafish human tumor xenograft model is that the optimal temperature for the development of zebrafish embryos is 28 °C^43^, whereas mammalian tumor cells grow optimally at 37 °C. A previous study has tested culture human tumor cells (leukemia cells) at different temperature (28 °C, 33 °C, 37 °C, 40 °C), and found that, when the cells were in exponential growth rate, the cells that cultured at 28 °C or 33 °C were in the “steady state of growth”, although a little slower than that cultured at optimal 37 °C^44^. Actually, for GBM tumor cells (U87 and U251) that were implanted in zebrafish, it has also been demonstrated that 28 °C and 35 °C incubation has no effect on the survival of cells after engraftment^45^. On the other hand, for the development of zebrafish embryos, it has been reported that they can tolerate up to 35 °C, although their survival is best at temperatures below 33 °C^43^ Thus, in some studies, the zebrafish mammalian tumor xenografts were kept at 28 °C^46,47^. In other cases, the culture temperature has been raised to 30 °C^45^ or even as high as 35 °C^48,49^.

Another possible limitation for the visual readout of BBB penetrating drugs in zebrafish larval is that the effective screening was limited to intrinsically fluorescent pharmaceuticals. Yet, it can also been solved, at least partly, by scanning the specific excitation spectra before testing them *in vivo* by confocal microscope, or designing specific marker tracer like DAPI to inject with the potential drug. In sum, in our study, we confirm that zebrafish orthotopic xenografted GBM model is an effective tool for studying the interaction of GBM tumor cells and local BBB, also representing a fast and cost-effective model-based approach for BBB penetrating anti-GBM drugs screening.

## Electronic supplementary material

Supplementary Information
