# Supplementary material for: Identify a Blood-Brain Barrier Penetrating Drug-TNB using Zebrafish Orthotopic Glioblastoma Xenograft Model
Source: Sci Rep. 2017 Oct 30;7:14372. doi: 10.1038/s41598-017-14766-2 (PMC5662771; doi:10.1038/s41598-017-14766-2)
Supplement: Supplementary file 1 — Supplementary Information [file 41598_2017_14766_MOESM1_ESM.pdf]

Identify a Blood-Brain Barrier Penetrating Drug-TNB using Zebrafish Orthotopic GBM Xenograft Model

Anqi Zeng, Tinghong Ye, Dan Cao, Xi Huang, Yu Yang, Xiuli Chen, Yongmei Xie, Shaohua Yao, Chengjian Zhao

Supplementary file contains:

3 supplementary figures;

Supplementary Figures:

Figure S1

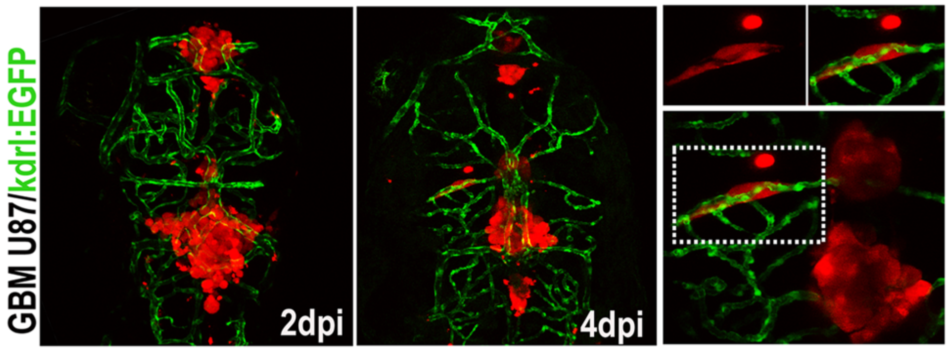

Figure S1: Living tracking of U87-RFP xenograft in flk1:EGFP zebrafish. Part of the area in 4dpi panel was magnified right, seperated channels of areas in dotted boxes were also showing upper.

Figure S2

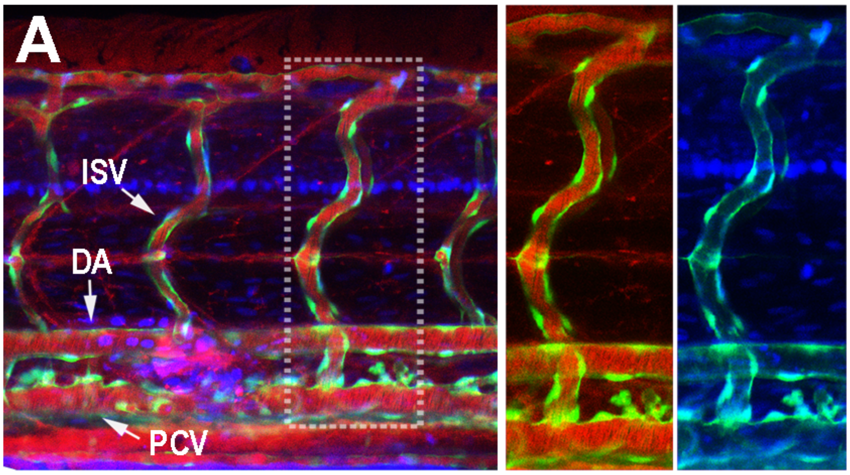

Figure S2: (A) The Dextran Texas Red (70,000 MW, 1mg/ml) and DAPI (350 MW, 1.5mg/ml) were mixed and injected into the circulation of 3dpf embryos (5nl/fish) from caudal vein (CV). Confocal living images of the segmental capillaries were obtained at 30 min after injection. (A) Both Dextran Texas Red and DAPI were leaked out from primary inter-segmental vessels (ISV) , dorsal aorta (DA) and post caudal vein (PCV). Areas in dotted boxes were magnified right.

Figure S3

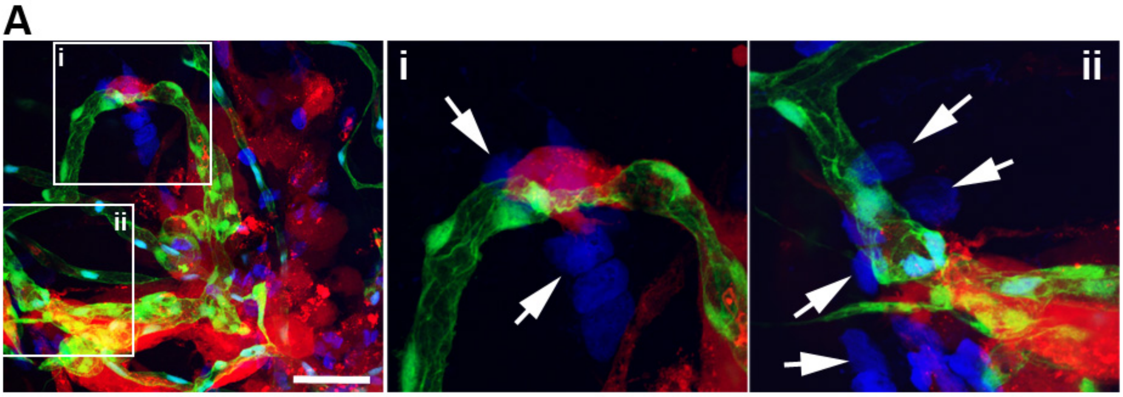

Figure S3: U87-RFP tumor cells were injected into the flk1: EGFP zebrafish brain as described in method section. DAPI was injected into the blood circulation though caudal vein 1 hour before the confocal imaging of the zebrafish brain. Two areas (i and ii) are magnified right, arrows indicate the DAPI leakage, scale bar indicates 100μm。
